# Extraction of Soybean and Pea Protein Isolates to Evaluate Therapeutic Potential Against Dexamethasone‐Induced Osteoporosis: In Vivo and *in Silico* Insights

**DOI:** 10.1002/fsn3.72068

**Published:** 2026-07-03

**Authors:** Hafiza Humaira Yasmeen, Adnan Amjad, Zafarullah Muhammad, Muhammad Tauseef Sultan, Khurram Afzal, Muhammad Israr, Ahmad Mujtaba Noman, Hassan Raza, Rabia Mehboob, Shehnshah Zafar, Entessar Mohammad Al Jbawi

**Affiliations:** ^1^ Faculty of Food Science and Nutrition Bahauddin Zakariya University Multan Punjab Pakistan; ^2^ College of Agriculture and Food Engineering Baise University Baise City Guangxi People's Republic of China; ^3^ Punjab Food Authority Lahore Punjab Pakistan; ^4^ Sugar Beet Research Department, Crop Research Administration General Commission for Scientific Agricultural Research (GCSAR) Damascus Syria

**Keywords:** inflammatory biomarkers, molecular docking, osteocalcin, osteoporosis, protein isolates

## Abstract

Soybean and pea protein isolates contain essential amino acid that may support bone health, skeletal function and muscle performance. The current research examines the amino acid profile of SPI and PPI and evaluates their impact on bone health in vivo and *in silico*. Female Sprague‐Dawley rats were randomly assigned to four groups: T_0_ (Standard diet + without osteoporosis), T_1_ (Standard diet + osteoporosis), T_2_ (2 g/kg bw of SPI+ 2 g/kg bw of PPI), and T_3_ (3 g/kg bw of SPI+ 3 g/kg bw of PPI). Biochemical parameters and histopathology of bone (femur and tibia), liver, and kidney were evaluated. The amino acid profile demonstrated higher levels of glutamic and aspartic acids and a lower level of sulfur‐containing amino acids. PPI exhibited higher content of arginine, lysine, and leucine than SPI. T_3_ significantly reduced the severity of disease compared with all other groups, as reflected in increased the osteocalcin levels (9.50 ± 1.85 ng/mL), Ca (9.10 ± 1.06 mg/dL), P (3.30 ± 0.08 mg/dL), and lowering inflammatory levels of CRP (0.70 ± 0.04 mg/L) and ESR (15.70 ± 1.58 mm/h) in the respective treatment groups. Histological studies revealed no changes in bone, liver, and kidney tissues. Furthermore, molecular docking demonstrated potential interactions between gamma‐carboxylase and ligands, such as genistein, β‐sitosterol, glycitein, kaempferol, and quercetin, with binding affinities of −8.3, −9.0, −7.6, −7.6, and −8.1 kcal/mol, respectively. These findings support the therapeutic potential of plant protein isolates in osteoporosis management; however, long‐term in vivo trials are required to validate the safety of isolates before moving toward human trials.

## Graphical abstract

(This graphical abstract demonstrates a comprehensive research study investigating the potential of soybean and pea protein isolates as therapeutic agents for osteoporosis, conducted through both in vivo (animal‐based) and in silico (computational) approaches).

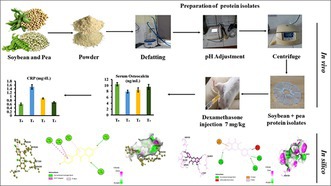

**Abbreviations**
ALP: alkaline phosphatase
ALT: alanine aminotransferase
AST: aspartate aminotransferase
Ca: serum calcium
CBC: complete blood count
CRP: C‐reactive protein
EDTA: ethylenediaminetetraacetic acid
ESR: erythrocyte sedimentation rate
GIOP: glucocorticoid‐induced osteoporosis
Hb: hemoglobin
HCT: hematocrit
MCH: mean corpuscular hemoglobin
MCHC: mean corpuscular hemoglobin concentration
MCV: mean corpuscular volume
MPV: mean platelet volume
OCN: osteocalcin
P: serum phosphorus
PDW: platelet distribution width
PLT: platelets
PPI: pea protein isolates
RANKL: receptor activator of nuclear factor kappa‐b ligand
RBC: red blood cells
SPI: soybean protein isolates
WBC: white blood cells

## 1 Introduction

Among bone diseases, osteoporosis is marked by bone loss and structural decline, elevating fracture risk, morbidity, and mortality, imposing a major burden on healthcare systems (Kong et al. 2025). The global prevalence is estimated at nearly 20%, rising sharply with age, while in Pakistan, more than 9.9 million people, predominantly women, are affected (Zheng et al. 2024; Akram et al. 2023; Nesar et al. 2024). A variety of factors contribute to osteoporosis, such as hormonal imbalance, immune dysfunction, aging, and long‐term use of glucocorticoids. GIOP occurs via suppression of osteoblastogenesis, promotion of osteoclast activity through RANKL/OPG imbalance, and increased oxidative stress (Rizzoli and Chevalley 2024).

The nutritional status and dietary protein play a crucial role in maintaining skeletal balance by enhancing Calcium (Ca) absorption and supporting bone matrix synthesis, whereas protein deficiency impairs bone metabolism and increases fracture risk (Yu et al. 2024). Many plants, including soybean and pea, are rich sources of protein (Rashwan et al. 2025), and are recognized as a sustainable intervention to support skeletal health. Soybean protein is notable for its balanced amino acid profile (e.g., like glutamic acid, leucine, and arginine) and bioactive isoflavones, such as genistein, daidzein, and glycitein, which interact with estrogen‐sensitive signaling pathways and have been linked to protection against metabolic and bone disorders (Tan et al. 2023; Nurmilah et al. 2024). Soybean protein isolates (SPI) are a complete plant‐based protein characterized by remarkable functional and physiological properties, primarily composed of β‐conglycinin (7S) and glycinin (11S), which together comprise nearly 70% of the total protein content (Lei et al. 2025).

The SPI has been shown to preserve bone health through multiple mechanisms. In obese rat models, SPI has been reported to support osteoblast‐related signaling, thereby preventing bone marrow lipid deposition and promoting osteoblast differentiation. Furthermore, SPI improves trabecular microarchitecture by reducing RANKL expression, thus suppressing osteoclast‐mediated bone resorption (Dirkes et al. 2018). It also protects against osteoblast senescence via activation of Sirt1 and modulation of the p53/p21 pathway (Gu et al. 2019). Pea seeds are highly valued for their rich nutritional content, which consists of high levels of protein, starch, fiber, and vital micronutrients (Castaldo et al. 2021). They also contain a wide range of phenolic compounds, including flavonoids (quercetin and kaempferol derivatives) and phenolic acids (gallic acid, ferulic, and p‐coumaric acids) (Shahidi and Yeo 2018). Furthermore, lignans and other polyphenols also boost their antioxidant activity (Sultan et al. 2023, 2014; Fahim et al. 2019). Pea‐derived bioactive components have begun to show promise in bone metabolism: for example, the tripeptide LRW, isolated from pea protein, stimulates osteoblastic proliferation, increases alkaline phosphatase, type 1 collagen, Runx2, and osteoprotegerin via Akt signaling, as well as enhancing matrix mineralization in MC3T3‐E1 cells (Arora et al. 2020).

While dietary pea protein supports muscle and protein retention, it is indirectly beneficial for skeletal strength (Salles et al. 2021). Pea protein isolates (PPI), known for their low allergenicity and numerous health benefits, are primarily composed of legumin (11S) and vicilin (7S) proteins. Bone remodeling and structural integrity are influenced by multiple factors, including hormonal changes, cytokines, and nutrient availability, all of which are strongly modulated by diet (Lei et al. 2025). These nutrients help in the prevention and control of diseases like such as arthritis, inflammation, high blood pressure, coronary heart disease, and cancer (Nasir et al. 2024; Shahidi and Yeo 2018). Despite increasing interest in plant proteins for bone health, most studies have evaluated SPI or PPI individually, while evidence on their combined use is scarce. The novelty of this study lies in combining SPI and PPI to demonstrate additive benefits in an osteoporosis model, with molecular docking providing exploratory insight into possible bioactive compound interactions.

The purpose of this study was to investigate the amino acid profiles of SPI and PPI and to assess the combined impact of SPI and PPI in an osteoporotic rat model. Specifically, it investigated the potential of these protein isolates to improve body weight and modulate feed and water intake. Additionally, the research examined the impact of SPI and PPI on CBC, liver enzymes and renal biomarkers, respectively. Furthermore, the influence of SPI and PPI on key serum biomarkers associated with bone health and inflammation, including serum osteocalcin, Ca, P, CRP, and ESR, was evaluated. Histopathological analysis of bone, liver, and kidney tissues was also conducted to evaluate tissue architecture and possible pathological changes. Overall, this study evaluated the impact of SPI and PPI on improving osteoporosis‐related physiological and biochemical changes and promoting bone health. The combined use of SPI and PPI appears beneficial for osteoporosis management; however, from a future perspective, more research in humans is needed to confirm their benefits and safety in reducing the risk of osteoporosis.

## 2 Materials and Methods

### 2.1 Procurement of Material

Distilled water, Sodium hydroxide (1.0 M NaOH), hexane, sulfuric acid (1.0 M H_2_SO_4_), deionized water, hydrochloric acid (HCl, 6 N), nitrogen gas (N_2_), performic acid, phenyl‐isothiocyanate (PITC), sodium acetate buffer, phosphate buffer acetonitrile (HPLC grade), methanol (HPLC grade), amino acid standard mixture, and all chemicals and reagents used in this research were purchased from GM Scientific Store, Iqbal Town, Multan. Soybean and pea seeds were purchased from the grain market in Multan, Pakistan. Seeds were washed, sun‐dried to eliminate unwanted materials. The particle size of the seeds was minimized to get a fine powder form using a grinder (Model No. SC250‐G) and stored in airtight zip bags. The preparation of protein isolates was performed in the Nutrition Lab, and the efficacy study was conducted at the Department of Human Nutrition, Bahauddin Zakariya University, Multan.

### 2.2 Preparation of Isolates

For SPI preparation, soybean powder (10 g) was defatted using a Soxhlet apparatus (Model number: CG 1368) with hexane (250 mL). The defatted powder was subsequently dried in a hot air oven (Model No. UNB 200) at 105°C for 10 min to ensure complete removal of hexane. After defatting, the powder was placed in a desiccator for 20 min and then stored in an airtight bag. Then, the defatted powder was dispersed in distilled water at (1:10, w/v). The pH of the dispersion was adjusted to 9.0 by the dropwise addition of 1.0 M NaOH, measured with a pH meter (Model No. ST 3100). The mixture was stirred at room temperature for 40 min using a magnetic stirrer (Model No. ZNCLB 1140), followed by centrifugation at 10,000 rpm for 20 min using a centrifuge (Model No. SIGMA 1–14) (Rout et al. 2024). Afterwards, the pH of the supernatant was reduced to 4.5 by the addition of 1.0 M H_2_SO_4_, followed by centrifugation for 10 min to obtain the protein precipitate (Xu et al. 2024). The obtained precipitate was washed with deionized water at (1:10, w/v), and the pH was adjusted to 7.0. Finally, the neutralized precipitate was then freeze‐dried and stored under refrigeration for further analysis (Wang et al. 2024). The preparation of PPI followed the same process as that of SPI.

### 2.3 Amino Acid Analysis

The amino acid composition of the protein isolates was analyzed following AOAC official methods. Briefly, the samples (20 mg) were subjected to acid hydrolysis with 6 N HCl solution (15 mL) in sealed glass tubes in a nitrogen atmosphere to prevent oxidation. To initiate hydrolysis the sample tubes were placed in a hot‐air oven (Model No. UNB 200) at 110°C for 20 h. For sulfur‐containing amino acids, the samples were subjected to pre‐oxidation with performic acid before hydrolysis, while tryptophan was analyzed separately using alkaline hydrolysis with sodium hydroxide (NaOH). After hydrolysis, the sample tubes were cooled to room temperature then filtered, and the solution was neutralized to pH of 6.5–7.0 using 6 N NaOH, measured with a pH meter (Model No. ST 3100). Then neutralized samples were centrifuged (Model No. ZNCLB 1140) at 4500 rpm for 5 min to remove insoluble particles. The clear supernatant was collected and, the samples were then derivatized with phenyl‐isothiocyanate (PITC), which is normally done in slightly alkaline conditions, i.e., at pH 9.0. The derivatized amino acids were separated and quantified using high‐performance liquid chromatography (HPLC) (Waters Corporation, Milford, MA, USA). The mobile phase consisted of sodium acetate or phosphate buffer and acetonitrile/methanol (HPLC grade) (Gorissen et al. 2018; Stone et al. 2015, and Custodio‐Mendoza et al. 2024).

### 2.4 Animal Model

In this study, 32 female Sprague–Dawley rats, weighing 100 to 110 g, were procured from the Department of Pharmacy, Bahauddin Zakariya University, Multan. The rats were housed under optimal conditions at a controlled temperature (22°C ± 3°C) with a 12‐h light/dark cycle. They were provided with a standard diet and had free access to tap water, following the protocol described by Saetang et al. (2024). The rats were randomly separated after a period of one week of acclimatization, each group containing eight rats. The groups and their respective treatments are presented in Table 1. The treatment groups received plant protein isolates via oral gavage once daily for 4 weeks. Throughout the study period, body weight, feed intake, and water intake were recorded weekly (Raza, Sultan, Noman, et al. 2025).

**TABLE 1:** Treatment plan.

Groups	Diets
Group 1	T_0_: Standard diet + no osteoporosis
Group 2	T_1_: Standard diet + osteoporosis
Group 3	T_2_: 2 g/kg bw of SPI+2 g/kg bw of PPI + osteoporosis
Group 4	T_3_: 3 g/kg bw of SPI+3 g/kg bw of PPI + osteoporosis

The doses used in the present study were chosen according to previously published safety studies of SPI and PPI in rodent models. Aouatifa et al. (2013) reported that no adverse effects following 90 days administration of PPI, with a no observed adverse effect level (NOAEL) of 8726 mg/kg bw/day in male rats and 9965 mg/kg bw/day in female rats. Similarly, Ali et al. (2015) reported that no adverse effects were observed when native (unmodified) SPI was fed to SD rats at 3333 mg/kg bw/day or pressurized SPI at 333–3333 mg/kg bw/day for 63 days. Furthermore, a 90‐day feeding study with genetically modified and non‐genetically modified soybeans revealed that at 1 g/kg/day and 5 g/kg/day, there were no biologically significant adverse effects in the diet (Lin et al. 2022). These findings support the safety of the doses selected for the present study.

#### 2.4.1 Induction of Disease

**FIGURE 1:**
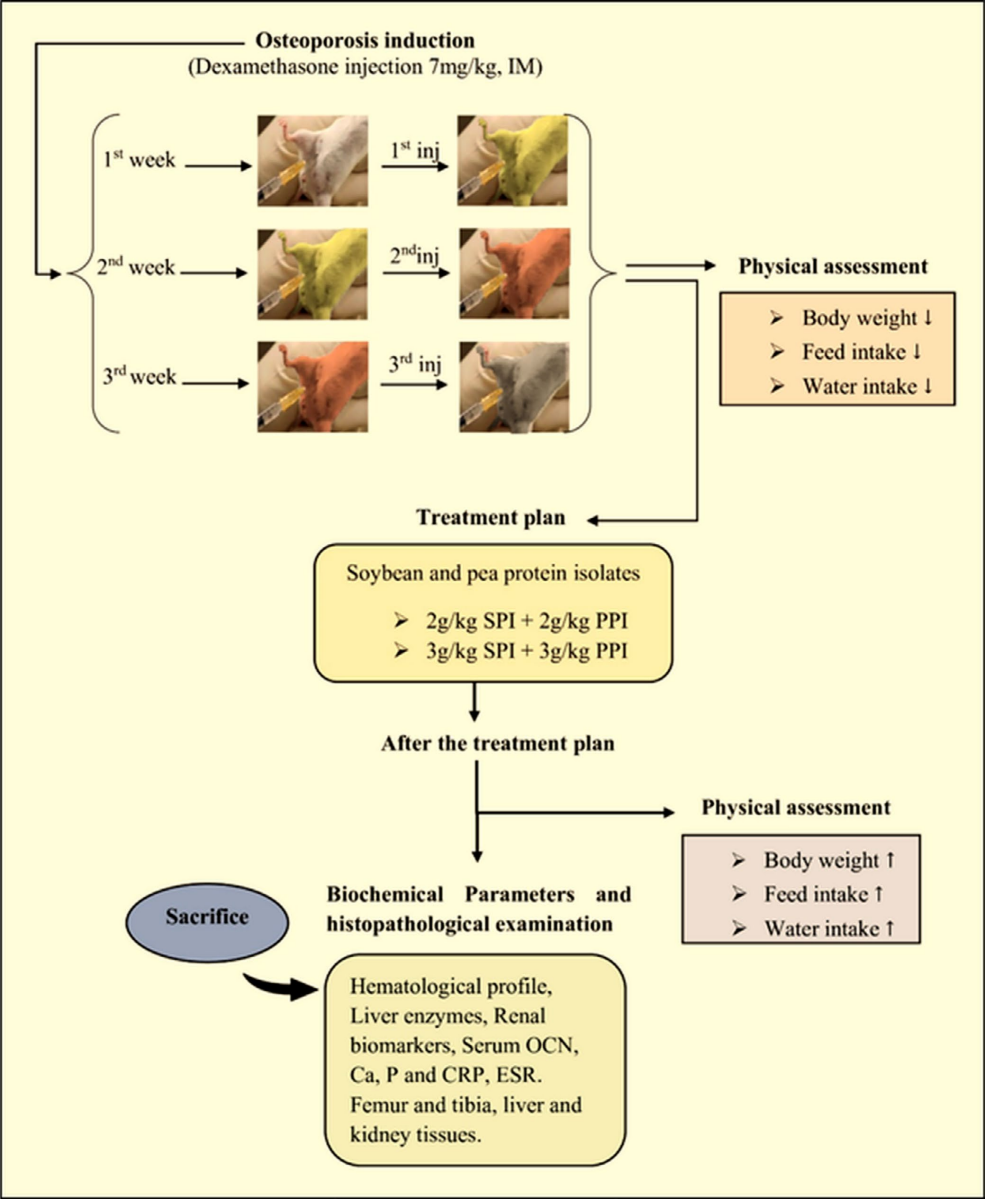
Study flow chart.

Osteoporosis was induced in all groups except the T_0_ group by intramuscular injection of dexamethasone sodium phosphate at a dose of 7 mg/kg, administered once weekly for 21 days. The dosage and protocol were selected based on previous studies to ensure effective induction of glucocorticoid‐related bone loss in rats. Following induction, rats were monitored daily (Badary et al. 2022; Hao et al. 2017). Figure 1 depicts the experimental flowchart of the study.

#### 2.4.2 Blood Sample and Tissue Collection

After 8 weeks of study, rats were anesthetized using an intraperitoneal injection of ketamine (80 mg/kg bw), and blood was collected into ethylenediaminetetraacetic acid (EDTA) and gel vials. Serum was separated from the gel vials by centrifugation at 4000 rpm for 10 min at room temperature using a centrifuge (Model No. CF0506). After centrifugation, the serum was transferred to Eppendorf tubes and maintained at −20°C until biochemical analysis (Saleh et al. 2024). After blood collection, the left femur and tibia were carefully cleaned and dissected of surrounding soft tissues. The liver and kidneys were also removed. All tissue samples were immediately fixed in 10% neutral‐buffered formalin for histopathological evaluation (Hao et al. 2017).

#### 2.4.3 Biochemical Analysis

Hematological parameters, such as RBC, Hb, HCT, MCH, MCV, MCHC, WBC, differential leukocyte counts, and platelet indices (MPV and PDW) were determined with an automated hematological analyzer (Sysmex K4500; Kobe, Japan). Liver function enzymes indices (ALT, AST and ALP) were determined by enzymatic colorimetric methods using commercially available diagnostic kits KHB‐ZY1280 (Shanghai Kehua, China) and total bilirubin was measured by spectrophotometry (Yu et al. 2022). Renal biomarkers such as serum creatinine, urea, Na, and K were analyzed using a Hitachi Roche p800 automated analyzer (Roche Diagnostics, USA) (Aparicio et al. 2014). Serum OCN levels were quantified using an enzyme‐linked immunosorbent assay (ELISA) kit (Sinogeneclon Biotec, China), while the concentration of serum Ca and P was estimated using BioSystems, S.A., diagnostic kits (Barcelona, Spain) (Badary et al. 2022). The Inflammatory biomarker CRP was quantified using a colorimetric assay kit (Elabscience Biotech Co., China), and ESR was determined by the Westergren method using EDTA‐anticoagulated whole blood (Nazeih et al. 2020).

### 2.5 Histopathological Analysis

The bone samples (femur and tibia) were fixed in nitric acid solution for 48 h after decalcification. The bone, liver, and kidney tissues were processed using standard histological methods, which involved dehydration in a graded series of alcohols, clearing in xylene, and embedding in paraffin wax. Tissue sections were cut into 4‐5 μm slices with a rotary microtome (RM225, Leica Biosystems, Germany) and stained with hematoxylin and eosin (H&E). Histopathological examination of tissues was done using an Olympus BX43 light microscope (Olympus Corporation, Tokyo, Japan) to evaluate the morphological changes and treatment‐related alterations in bone, liver and kidney tissues (Badary et al. 2022).

### 2.6 Ethical Approval

The study was conducted in strict accordance with the ethical principles for animal research as outlined in the ARRIVE (Animal Research: Reporting of In Vivo Experiments) guidelines. The ethical approval for this research was granted by the Institutional Animal Ethics Committee (IAEC), Department of Human Nutrition (DoHN), Faculty of Food Science and Nutrition (FFSN), Bahauddin Zakariya University (BZU), Multan, Pakistan, under Letter No. FFSN, DoHN#13334–25. All experimental procedures, animal handling, and housing conditions complied with internationally accepted standards for the care and use of laboratory animals.

### 2.7 Molecular Docking Analysis

Molecular docking utilizes *in silico* techniques to estimate the most favorable positioning and interaction strength between a ligand and its target protein. It simulates intermolecular interactions and explores different conformations to identify potential therapeutic agents (Vidal‐Limon et al. 2022). The current section describes the molecular docking of five bioactive compounds from soybean and pea, namely, glycitein, genistein, kaempferol, β‐sitosterol, and quercetin, against gamma‐carboxylase to evaluate osteoporosis role of soybean and pea. The ligands, i.e., glycitein (CID 5317750), genistein (CID 5280961), kaempferol (CID 5280863), β‐sitosterol (CID 222284), and quercetin (CID 5280343) were accessed in 3D SDF format from the PubChem database. The protein gamma‐carboxylase (PBD ID 9bvp) was obtained from the Protein Data Bank (PDB). Furthermore, binding affinity was analyzed using PyRx (AutoDock Vina 1.1.2), and molecular docking interactions were visualized through Discovery Studio (version 21.1.020298) (Raza, Sultan, Ahmad, et al. 2025).

### 2.8 Statistical Analysis

The mean ± standard deviation (SD) was used to express all the obtained results. Data analysis was performed using Statistical Software, version 8.1 (StatSoft Inc., USA). Treatment effects in a factorial design were evaluated using two‐way analysis of variance (ANOVA). Significant differences among groups were considered statistically significant at *p* < 0.05 (Eze and Ortutu 2024).

## 3 Results

### 3.1 Amino Acid Composition of Soy Protein Isolates and Pea Protein Isolates

The amino acid composition of SPI and PPI is presented in Table 2. Glutamic and aspartic acid were highest in both SPI (17.1%, 10.13%) and PPI (16.5%–18%, 10.5–11.5), while sulfur‐containing amino acids, methionine and cysteine, were slightly lower. Moreover, PPI showed higher levels of arginine (8.0%–8.8%), lysine (6.8%–7.5%), and leucine (7.0%–7.8%) as compared to SPI arginine (6.6%), lysine (5.4%), and leucine (6.76%), respectively. Tryptophan levels ranged from 1.21% in SPI to 0.9%–1.2% in PPI. Overall, both protein isolates exhibited a balanced amino acid composition; however, PPI showed a slightly higher content of some essential amino acids such as arginine, lysine, and leucine.

**TABLE 2:** Amino acid composition of soy protein isolates and pea protein isolates.

Amino acids	Soy protein isolates (%)	Pea protein isolates (%)
Glutamic acid	17.1	16.5–18.0
Aspartic acid	10.13	10.5–11.5
Leucine	6.76	7.0–7.8
Arginine	6.6	8.0–8.8
Lysine	5.4	6.8–7.5
Phenylalanine	4.57	4.5–5.2
Proline	4.57	4.0–4.8
Serine	4.35	4.5–5.2
Valine	4.12	4.5–5.0
Isoleucine	4.09	4.0–4.6
Alanine	3.67	4.0–4.6
Glycine	3.56	3.8–4.5
Threonine	3.48	3.5–4.0
Tyrosine	3.48	3.2–3.8
Histidine	2.2	2.4–2.8
Tryptophan	1.21	0.9–1.2
Methionine	1.0	0.8–1.2
Cysteine	1.0	0.9–1.3

### 3.2 Effects of SPI and PPI Mixture on Rats' Feed and Water Intake

**FIGURE 2:**
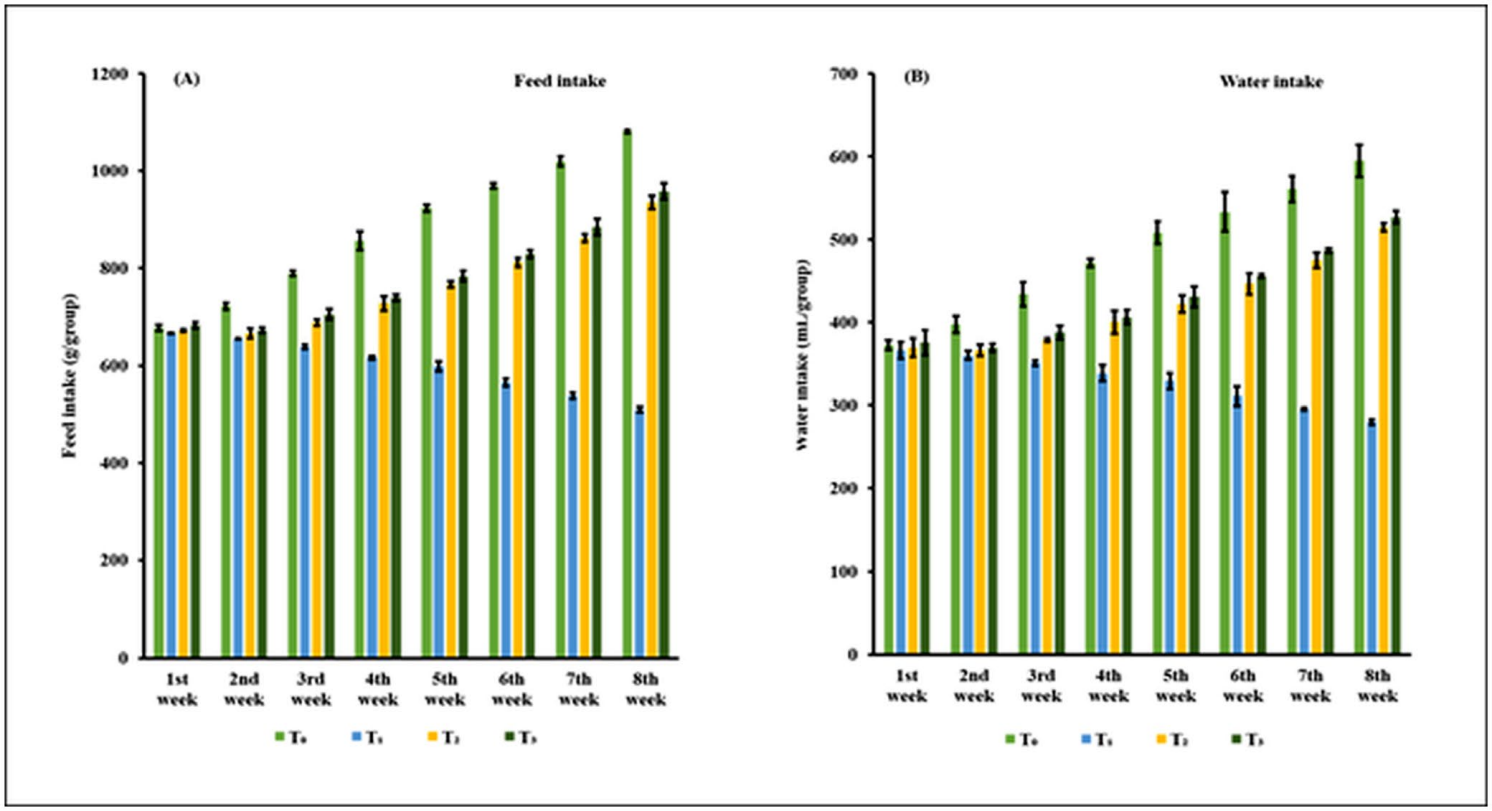
Effects of SPI and PPI mixture on feed and water intake in rats (A) Feed intake; (B) Water intake. T_0_: (Standard diet + no osteoporosis); T_1_: (Standard diet + osteoporosis); T_2_: (2 g/kg of SPI + 2 g/kg of PPI + osteoporosis); T_3_: (3 g/kg of SPI + 3 g/kg of PPI + osteoporosis). Data values were expressed in mean ± SD.

The intake of feed and water in rats across different groups during the 8 week study is presented in Figure 2. The results indicate that a consistent decrease in feed and water consumption occurred throughout the study in T_1_ compared with T_0_; however, feed and water consumption improved in the treatment groups. Meanwhile, the T_2_ and T_3_ groups demonstrated a significant improvement in feed and water intake. Particularly, the T_3_ group recorded the highest feed and water intake compared with T_1_ and was closer to T_0_.

### 3.3 Effects of SPI and PPI Mixture on Rats' Body Weight

**FIGURE 3:**
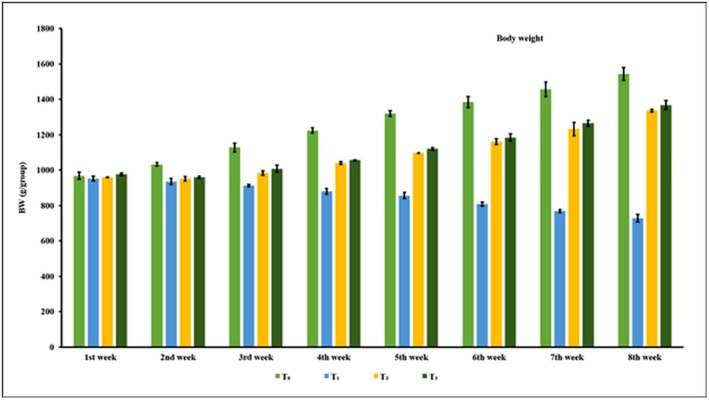
Effects of SPI and PPI mixture on the rat's body weight. (T_0_) Standard diet + no osteoporosis, (T_1_) Standard diet + osteoporosis, (T_2_) 2 g/kg of SPI + 2 g/kg of PPI + osteoporosis, (T_3_) 3 g/kg of SPI + 3 g/kg of PPI + osteoporosis. Data values were expressed in mean ± SD.

The rats' body weight was measured every week for during the of 8‐week study and is shown in Figure 3. The body weight was reduced in T_1_ compared with T_0_, but it improved in the treatment groups. Notably, rats in the T_3_ group exhibited a significant improvement in weight gain.

### 3.4 Effects of SPI and PPI Mixture on Hematological Profile

Table 3 shows the analysis of the hematological profile, revealing statistically significant differences (*p* < 0.05) between the groups. The values of RBC and Hb were significantly reduced in T_1_, but a slight improvement was observed in the treatment groups, particularly in T_3_, with values of (6.10 ± 0.67 10^6^/μL) and (12.30 ± 1.62 g/dL), respectively. HCT% values improved in the treated group but lower in T_1_ group, when compared to the T_0_ group, but improved in the treated group. Maximum values were recovered in T_3_ (39.40% ± 2.50%). However, WBC was significantly higher in T_1_ (7.20 ± 2.21 10^3^/μL) than in T_0_, but T_3_ exhibited a slight decrease in WBC count (6.40 ± 1.23 10^3^/μL). Meanwhile, the monocyte counts were significantly reduced in T_3_ than in T_0_ and T_1_. Eosinophil and basophil counts improved in T_3_ compared with T_1_. The platelet count was highest in the T_1_ (326.00 ± 12.95 10^3^/μL) compared to T_0,_ whereas T_3_ showed intermediate levels (275 ± 22.22 10^3^/μL). MPV and PDW values were lower in T_1_ than in T_0,_ and the greatest improvement was observed in T_3_.

**TABLE 3:** Effect of SPI and PPI mixture on hematological profile.

Treatments	T_0_	T_1_	T_2_	T_3_
RBCs (10^6^/μL)	6.40 ± 0.78^a^	5.70 ± 0.49^a^	5.90 ± 0.59^a^	6.10 ± 0.67^a^
Hb (g/dL)	12.60 ± 1.20^a^	11.40 ± 1.60^a^	11.90 ± 1.76^a^	12.30 ± 1.62^a^
HCT 8 (%)	39.20 ± 2.06^a^	34.50 ± 2.59^b^	36.10 ± 2.42^ab^	39.40 ± 2.50^a^
MCV (fL)	61.25 ± 4.83^a^	60.52 ± 5.72^a^	61.18 ± 5.87^a^	64.59 ± 4.43^a^
MCH (pg)	19.68 ± 1.61^a^	20.00 ± 1.27^a^	20.16 ± 1.40^a^	20.16 ± 2.65^a^
MCHC (g/dL)	32.14 ± 1.72^a^	33.04 ± 1.16^a^	32.96 ± 0.56^a^	31.21 ± 2.25^a^
WBCs (10^3^/μL)	4.80 ± 1.36^c^	7.20 ± 2.21^a^	6.40 ± 1.23^b^	5.90 ± 1.01^c^
Lymphocytes (%)	53.40 ± 2.70^a^	56.10 ± 1.94^a^	55.70 ± 1.81^a^	54.40 ± 3.11^a^
Monocytes (%)	0.10 ± 0.03^c^	0.89 ± 0.05^a^	0.71 ± 0.06^b^	0.58 ± 0.02^c^
Neutrophil (%)	43.40 ± 3.96^a^	40.20 ± 2.052^a^	40.20 ± 4.90^a^	41.70 ± 2.50^a^
Eosinophil (%)	0.12 ± 0.004^d^	0.23 ± 0.001^a^	0.19 ± 0.01^b^	0.15 ± 0.12^c^
Basophil (%)	2.50 ± 0.12^c^	3.37 ± 0.23^a^	3.02 ± 0.14^ab^	2.94 ± 0.36^b^
PLT (10^3^/μL)	253 ± 12.99^c^	326 ± 12.95^a^	297 ± 16.23^ab^	275 ± 22.22^bc^
MPV (fL)	9.18 ± 0.50^a^	6.80 ± 0.36^b^	5.90 ± 0.31^b^	6.50 ± 0.49^b^
PDW (fL)	14.90 ± 1.57^b^	10.70 ± 1.63^c^	16.50 ± 1.81^ab^	18.10 ± 2.57^a^

*Note:* T_0_: (Standard diet + no osteoporosis), T_1_: (Standard diet + osteoporosis), T_2_: (2 g/kg of SPI + 2 g/kg of PPI + osteoporosis), T_3_: (3 g/kg of SPI + 3 g/kg of PPI + osteoporosis). Data values were expressed in mean ± SD. a, b, c and d letters show level of significance among the treatments. Similar letters indicate no significance while different letters indicate significance level among treatments.

### 3.5 Effects of SPI and PPI Mixture on Liver Enzymes

**FIGURE 4:**
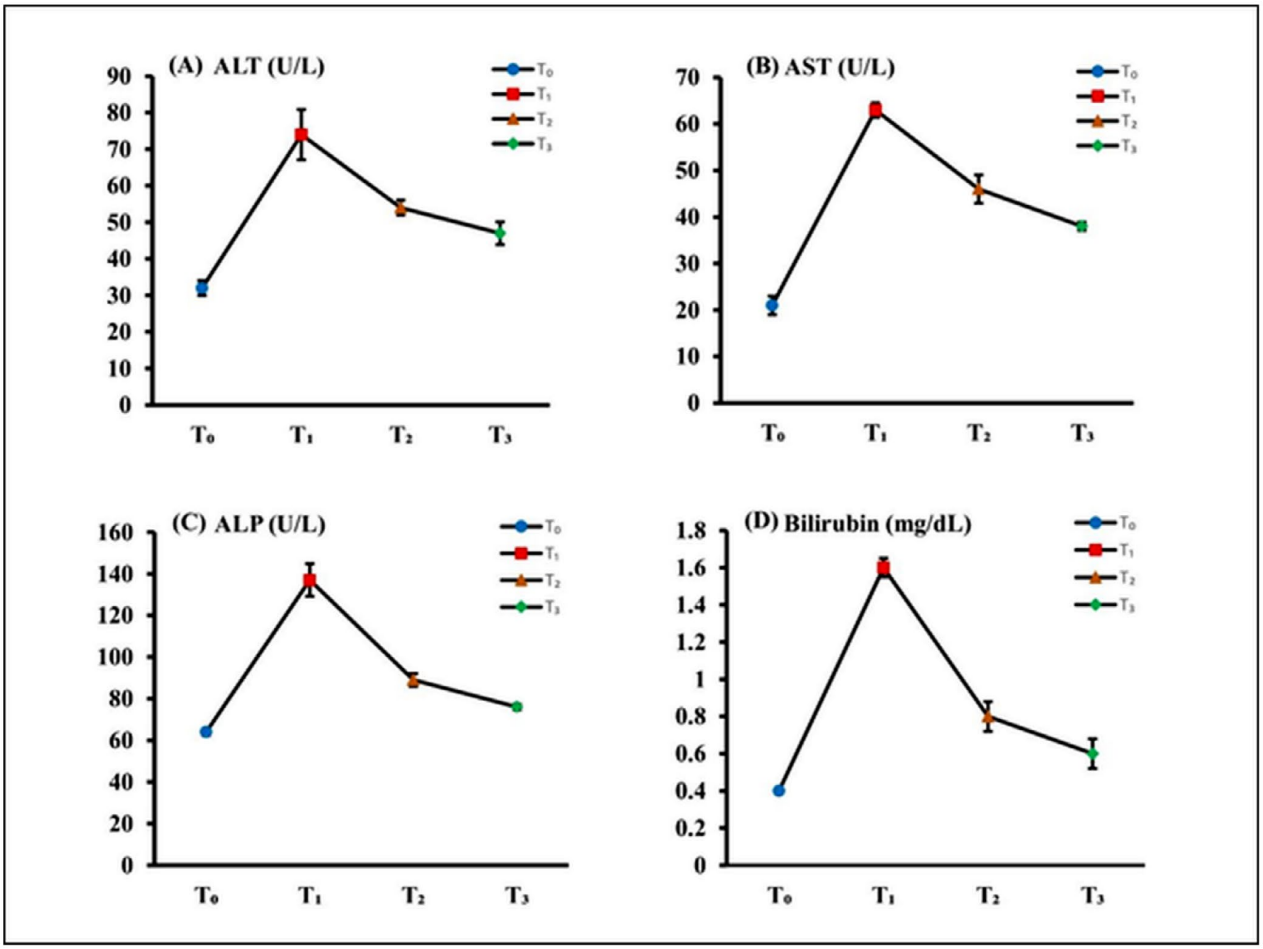
Effect of SPI and PPI mixture on liver enzymes (A) ALT, (B) AST, (C) ALP, and (D) Bilirubin. T_0_: (Standard diet + no osteoporosis), T_1_: (Standard diet + osteoporosis), T_2_: (2 g/kg bw of SPI + 2 g/kg bw of PPI + osteoporosis), T_3_: (3 g/kg bw of SPI + 3 g/kg bw of PPI + osteoporosis). Data values were expressed in mean ± SD.

Significant (*p* < 0.05) variation was noted among the treatment groups for all liver enzyme indices (ALT, AST, ALP, and bilirubin), as illustrated in Figure 4. The results depicted that the levels of liver enzymes were elevated in T_1_ as compared with T_0,_ but the levels were improved in T_3_. Meanwhile, all liver enzyme parameters were increased in T_1_, with recorded values of (74.00 ± 6.86 U/L), (63.00 ± 1.56 U/L), (137.00 ± 7.84 U/L), and (1.60 ± 0.05 mg/dL), respectively, but were significantly reduced in the treatment groups. The greatest improvement was noted in T_3,_ with values of (47.00 ± 3.08 U/L), (38.00 ± 0.87 U/L), (76.00 ± 1.24 U/L), and (0.60 ± 0.07 mg/dL).

### 3.6 Effects of SPI and PPI Mixture on Renal Biomarkers

**FIGURE 5:**
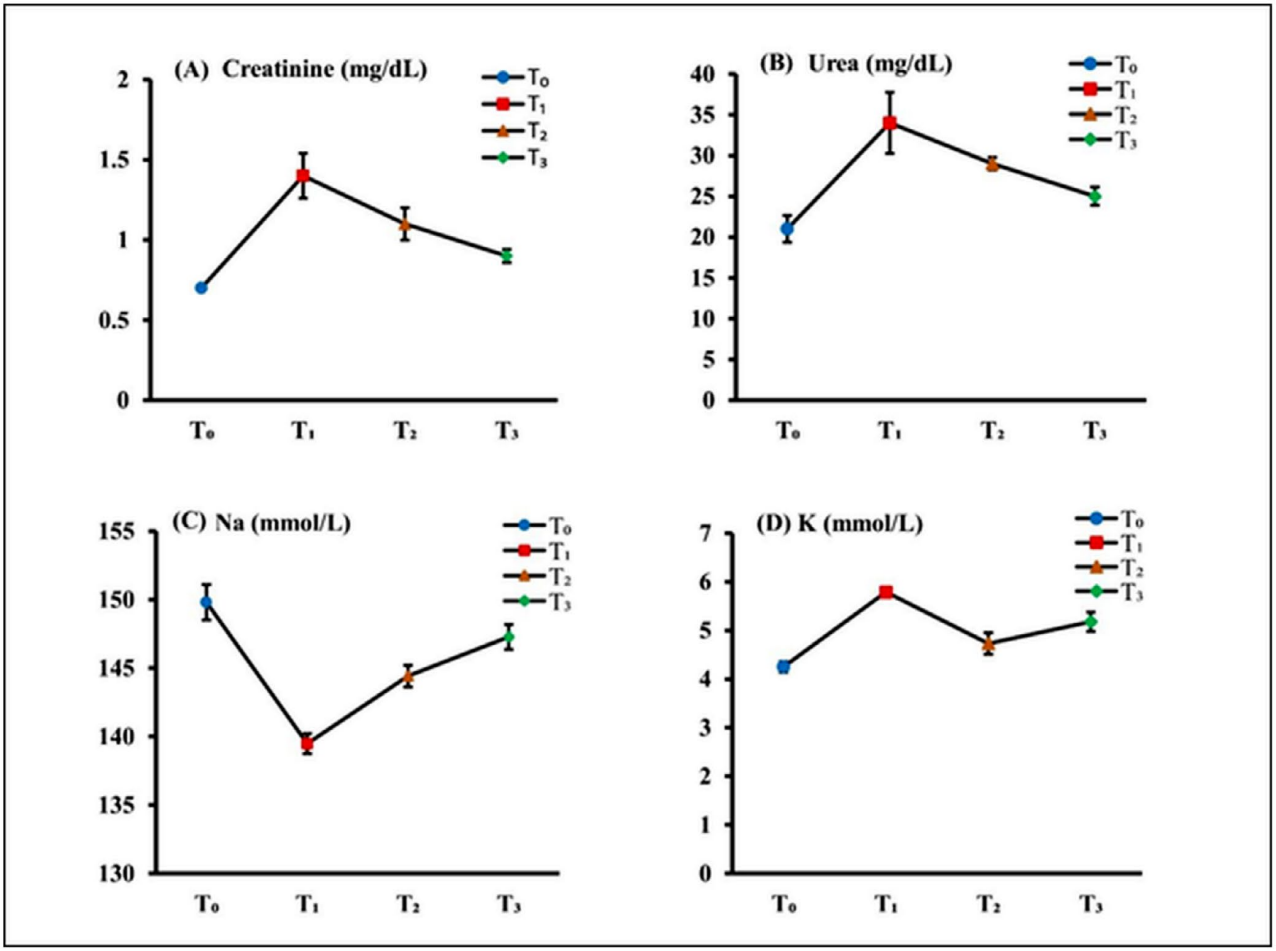
Effect of SPI and PPI mixture on renal biomarkers: (A) Creatinine, (B) Urea, (C) Na, and (D) K. T_0_: (Standard diet + no osteoporosis), T_1_: (Standard diet + osteoporosis), T_2_: (2 g/kg bw of SPI + 2 g/kg bw of PPI + osteoporosis), T_3_: (3 g/kg bw of SPI + 3 g/kg bw of PPI + osteoporosis). Data values were expressed in mean ± SD.

Figure 5 represents the significant results shown in all parameters of renal biomarkers, such as creatinine, urea, Na, and K levels among the groups (*p* < 0.05). Creatinine and urea levels were significantly increased in T_1_ when compared with T_0_, but improved in the treated groups. Meanwhile, a significant reduction was observed in T_3_, with values of (0.90 ± 0.048 mg/dL and 25.00 ± 1.11 mg/dL), indicating renal biomarker recovery. Moreover, Na concentration was lowest in T_1_ as compared with T_0_, but it improved in the treated groups, particularly T_3,_ with intermediate values (147.28 ± 7.91 mmol/L), respectively. Although T_1_ showed the highest K value (5.79 ± 0.09 mmol/L) as compared with T_0_, T_3_ (5.18 ± 0.20 mmol/L) showed values comparable to the control.

### 3.7 Effects of SPI and PPI Mixture on Serum Biomarkers and Inflammatory Markers

**FIGURE 6:**
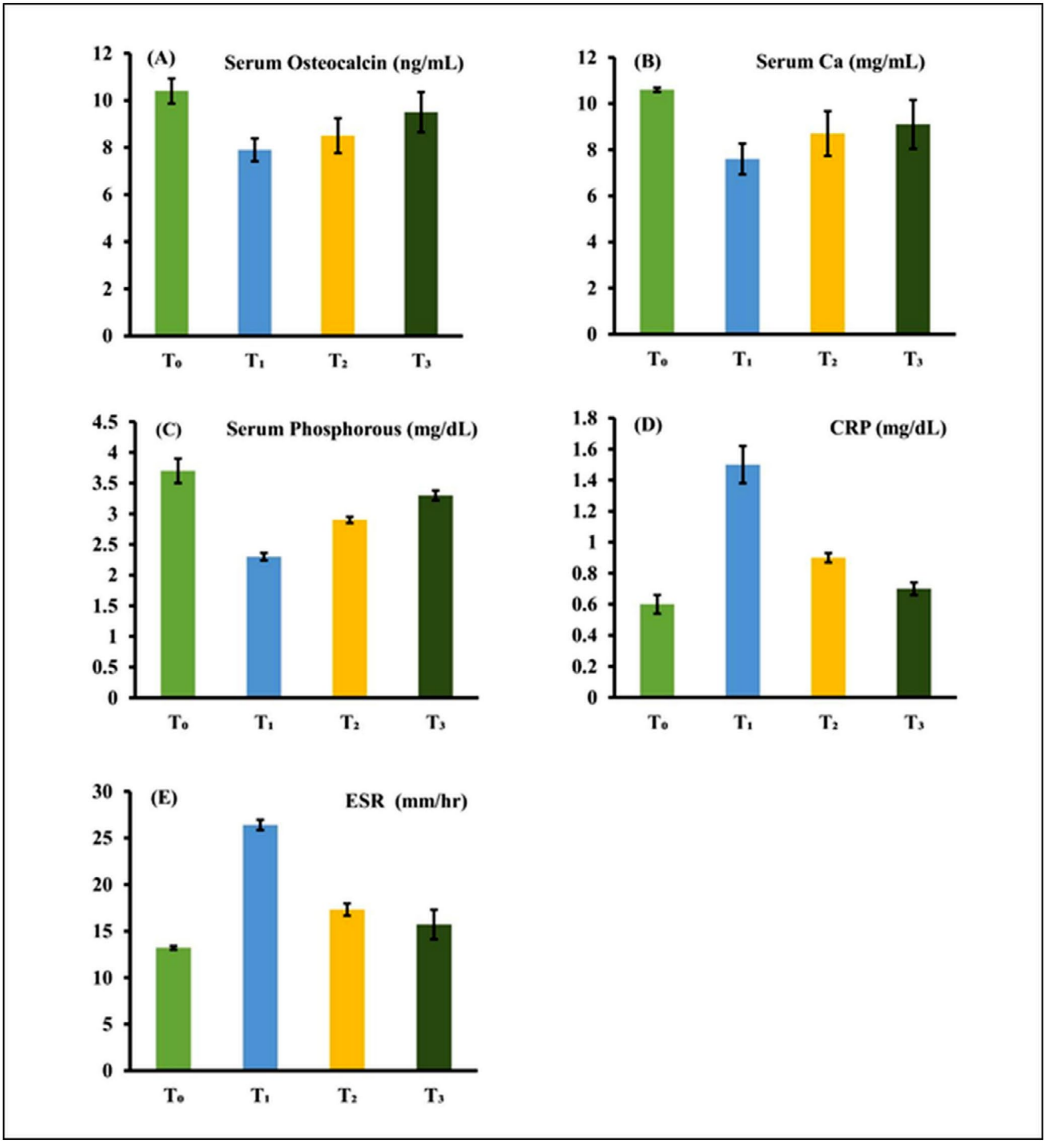
Effects of SPI and PPI mixture on serum biomarkers (A) Osteocalcin, (B) Calcium, and (C) Phosphorus, and inflammatory markers (D) CRP and (E) ESR. T_0_: (standard diet + no osteoporosis), T_1_: (standard diet + osteoporosis), T_2_: (2 g/kg bw of SPI + 2 g/kg bw of PPI + osteoporosis), T_3_: (3 g/kg bw of SPI + 3 g/kg bw of PPI + osteoporosis). Data values were expressed as mean ± SD.

The results for serum osteocalcin, Ca, P, CRP and ESR (*p* < 0.05) are presented in Figure 6. The results depicted that the levels of serum osteocalcin, Ca, and P were significantly decreased in T_1_ as compared with T_0_ (7.90 ± 1.48 ng/mL; 7.60 ± 0.67 mg/dL and 2.30 ± 0.06 mg/dL), but the levels improved in T_3_ (9.50 ± 1.85 ng/mL; 9.100 ± 1.06 mg/dL, and 3.30 ± 0.08 mg/dL) indicating a restorative effect on osteoblastic activity. However, CRP and ESR levels were significantly raised in T_1_ (1.50 ± 0.12 mg/dL and 26.40 ± 0.55 mm/h) as compared to T_0_, but T_3_ showed a significant reduction in values (0.70 ± 0.04 mg/dL and 15.70 ± 1.58 mm/h) due to the therapeutic effect of the treatment.

### 3.8 Effects of SPI and PPI Mixture on Histological Alterations in Rat Femur and Tibia Tissues

**FIGURE 7:**
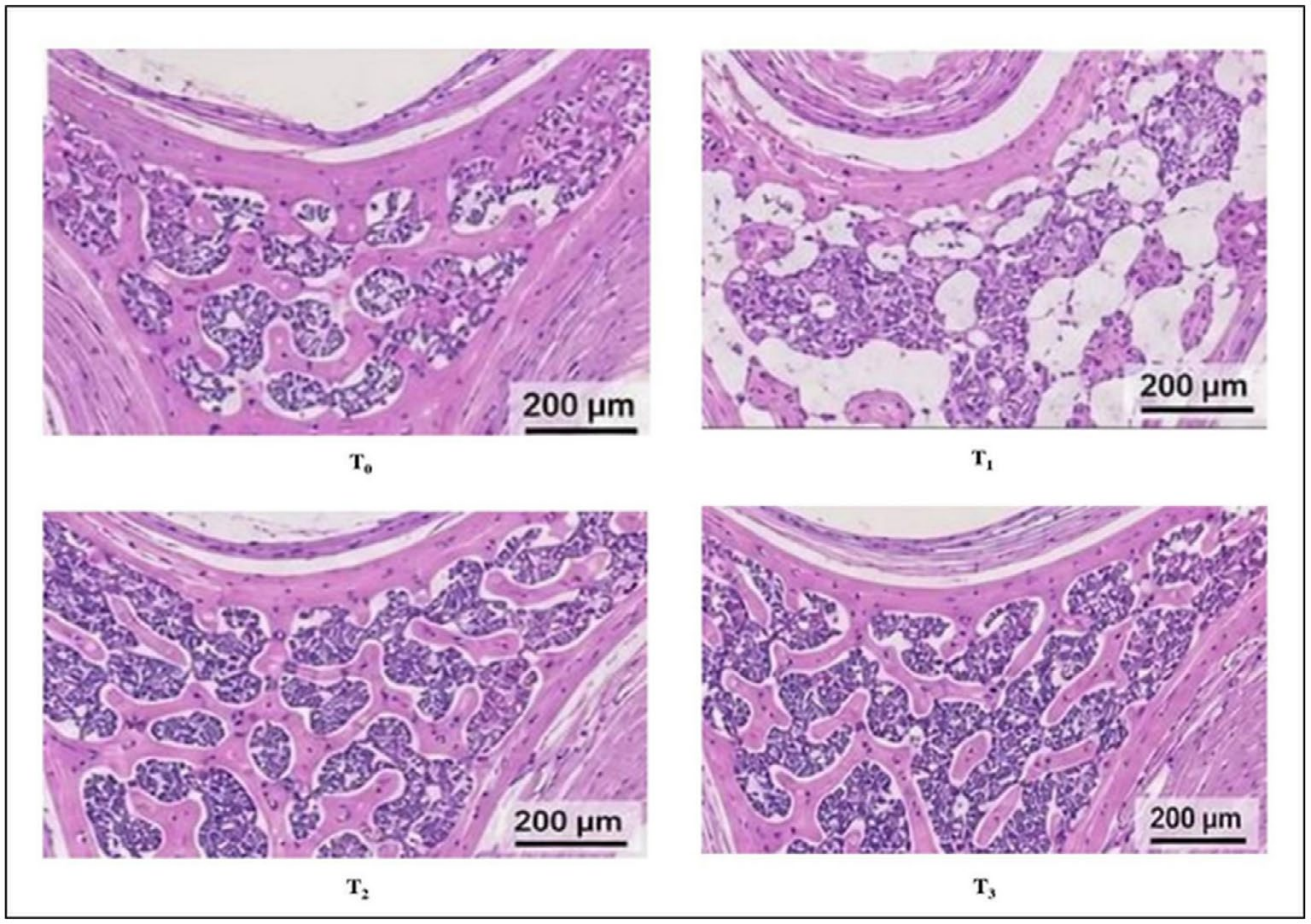
Histopathological examination of femur tissues from experimental rats. T_0_: (Standard diet + no osteoporosis), T_1_: (Standard diet + osteoporosis), T_2_: (2 g/kg bw of SPI + 2 g/kg bw of PPI + osteoporosis), T_3_: (3 g/kg bw of SPI + 3 g/kg bw of PPI + osteoporosis).

The histopathological changes in the femur and tibia tissues of the experimental groups are shown in Figure 7. The histological sections of the femur showed well‐organized trabecular bone and normal cellular arrangement in T_0_. In T_1_, there were marked changes (severe) in the trabeculae which were sparse, porous with enlarged intertrabecular spaces. The treatment groups (T_2_ and T_3_) exhibited a significant improvement in the structure of the femur, including enhanced trabecular connectivity and increased bone matrix formation. T_3_ resulted in a near‐normal histological appearance, reflecting the successful reduction of femural degeneration due to osteoporosis.

**FIGURE 8:**
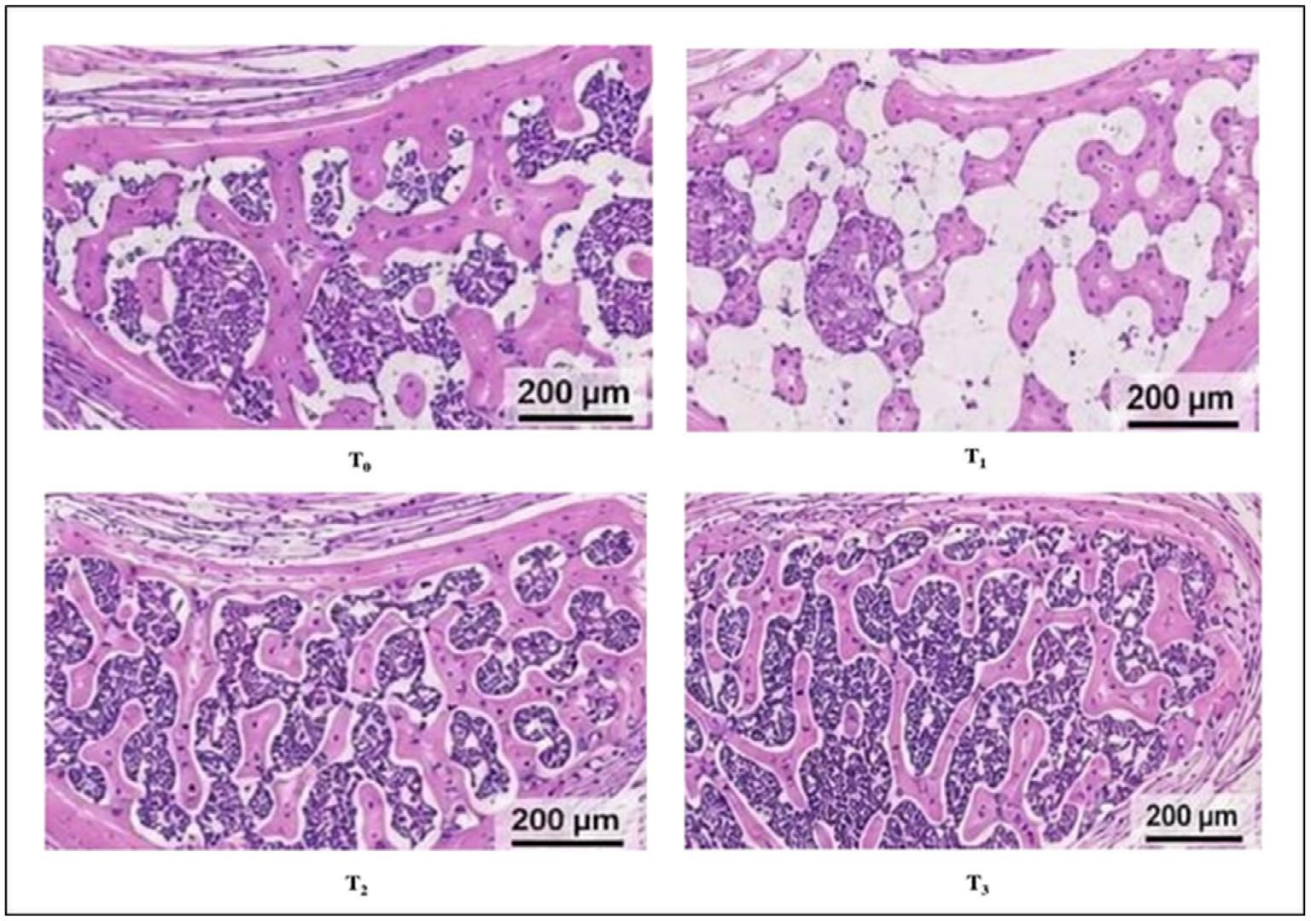
Histopathological examination of tibia tissues from experimental rats. (T_0_) (Standard diet + no osteoporosis), (T_1_) (Standard diet + osteoporosis), (T_2_) (2 g/kg bw of SPI + 2 g/kg bw of PPI + osteoporosis), (T_3_) (3 g/kg bw of SPI + 3 g/kg bw of PPI + osteoporosis).

The result of the histopathological examination is presented in Figure 8. In T_0_, the histopathology of the tibia showed a normal, compact trabecular bone structure, thus healthy bone architecture. T_1_ demonstrated significant changes such as increased marrow space, decreased trabecular thickness, and decreased bone density, which are characteristics of osteoporosis. The bone microstructure had significant improvement in the treatment groups T_2_ and T_3_ compared with the osteoporotic group, with an increase in trabecular thickness and a decrease in marrow cavity size. The restorative effect was more evident in T_3_, suggesting that the high dose of the SPI and PPI mixture had a positive effect on the integrity of the tibial bone, and less osteoporotic damage was observed.

### 3.9 Effects of SPI and PPI Mixture on Histological Alterations in Rat Liver Tissues

**FIGURE 9:**
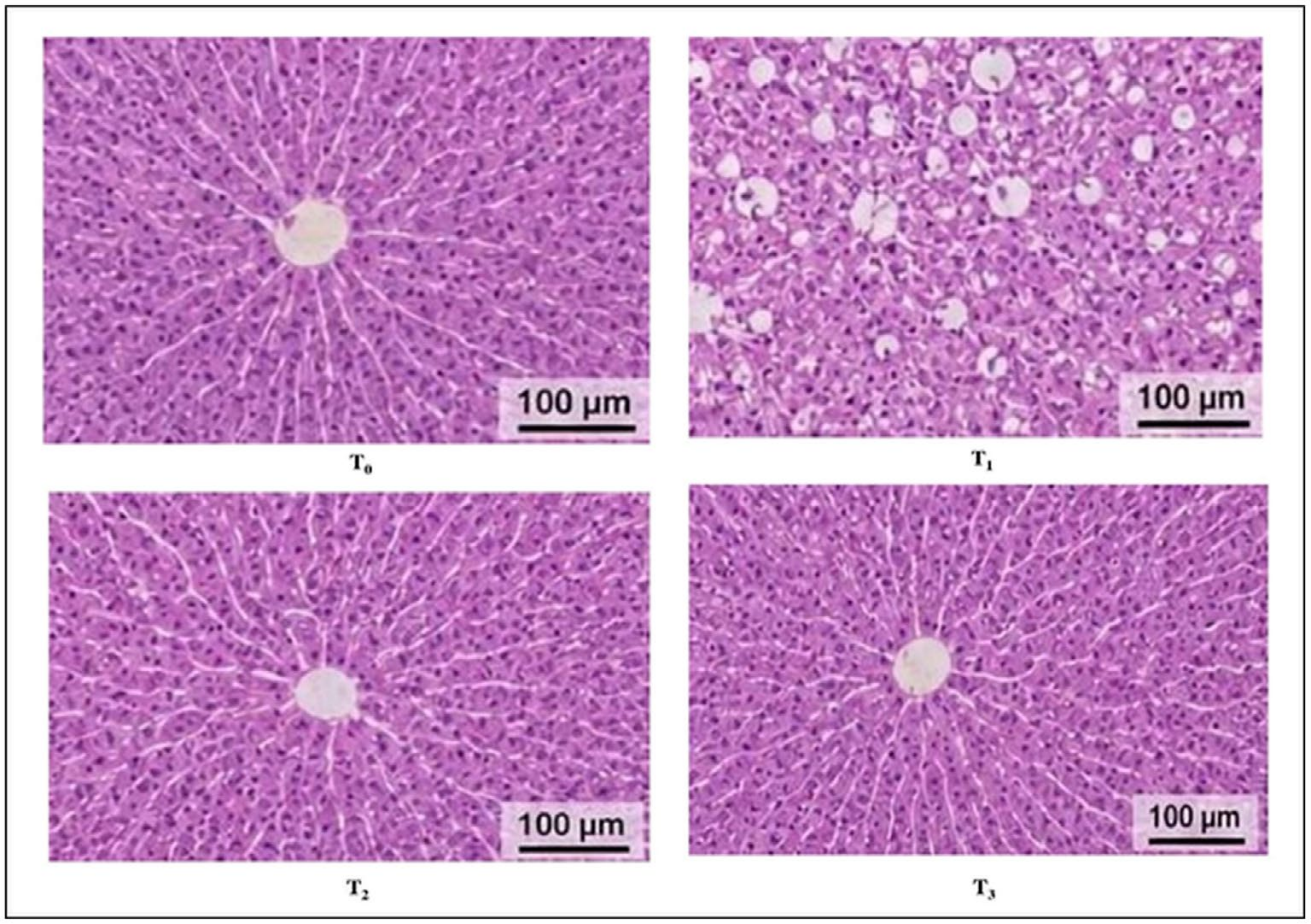
Histopathological examination of liver tissues from experimental rats. T_0_: (Standard diet + no osteoporosis), T_1_: (Standard diet + osteoporosis), T_2_: (2 g/kg bw of SPI + 2 g/kg bw of PPI + osteoporosis), T_3_: (3 g/kg bw of SPI + 3 g/kg bw of PPI + osteoporosis).

Figure 9 depicts the microscopic changes in liver tissues. In T_0_, the liver areas exhibited normal hepatic cords, central veins, and normal hepatocyte arrangement. Histological results of T_1_ showed hepatocellular vacuolation, fatty degeneration, and liver disorganization, which are all liver stress responses associated with osteoporosis condition. In T_2_ and T_3_ showed marked improvement in liver histology was observed, with improved hepatocyte arrangement, decreased vacuolation, and restoration of normal hepatic architecture. The protective effects were more observed in T_3_, indicating that a high dose mixture of SPI and PPI had positive effects on the liver and reduced damage to the tissues.

### 3.10 Effects of SPI and PPI Mixture on Histological Alterations in Rat Kidney

**FIGURE 10:**
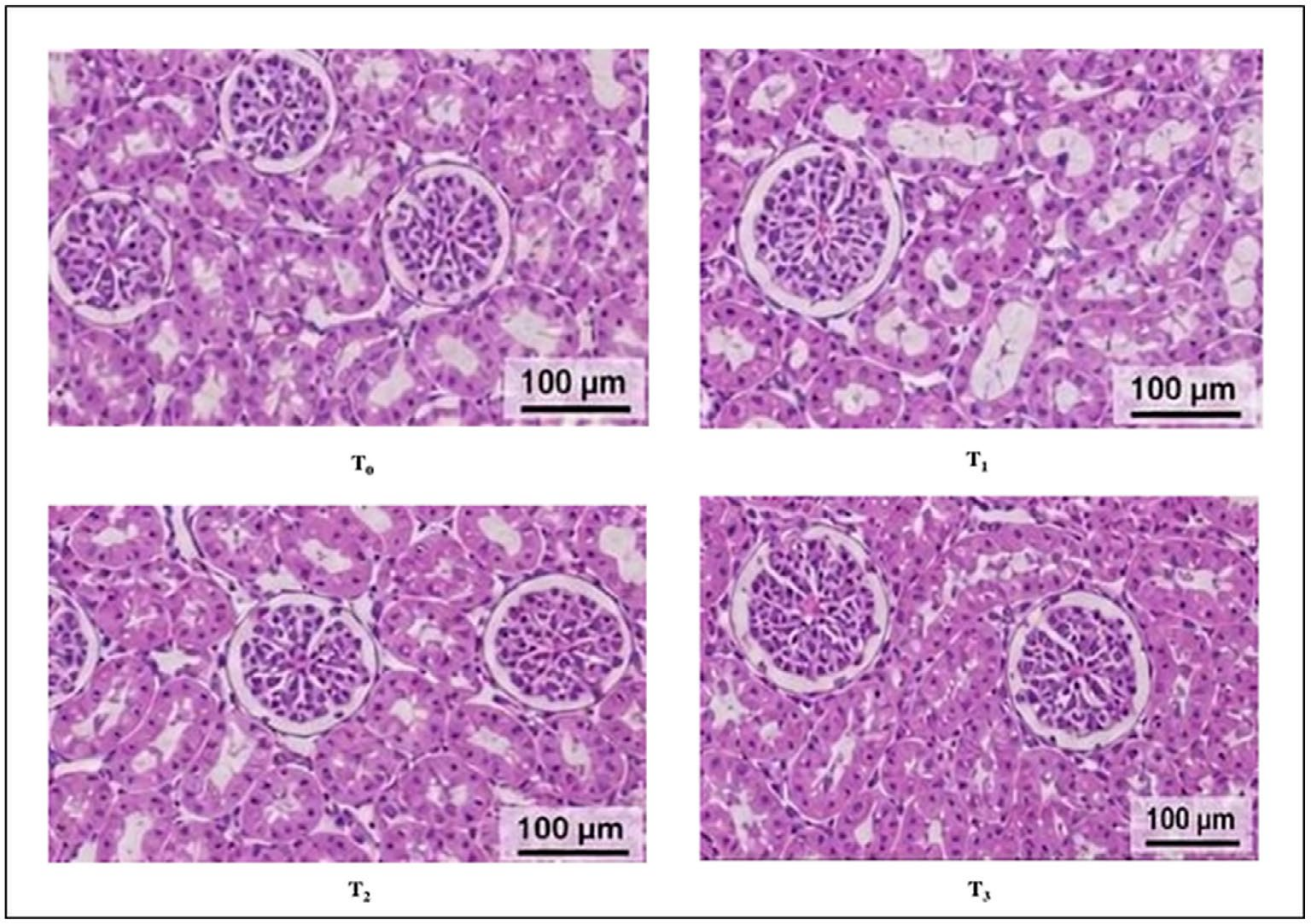
Histopathological examination of kidney tissues from experimental rats. T_0_: (Standard diet + no osteoporosis). T_1_: (Standard diet + osteoporosis). T_2_: (2 g/kg bw of SPI + 2 g/kg bw of PPI + osteoporosis). T_3_: (3 g/kg bw of SPI + 3 g/kg bw of PPI + osteoporosis).

Histology of the kidneys of T_0_ revealed normal renal corpuscles and tubular structures with no pathological changes presented in Figure 10. In T_1_, mild renal damage was observed, along with tubular degeneration, cellular swelling, and some disruption of glomerular architecture, which was believed to be the result of metabolic stress caused by osteoporosis. T_2_ and T_3_ had better renal morphology and normal glomeruli, with minimal tubular damage. These results clearly show that neither T_2_ nor T_3_ treatment is toxic to renal tissues and may support the renal tissue integrity.

### 3.11 Molecular Docking Insights

**FIGURE 11:**
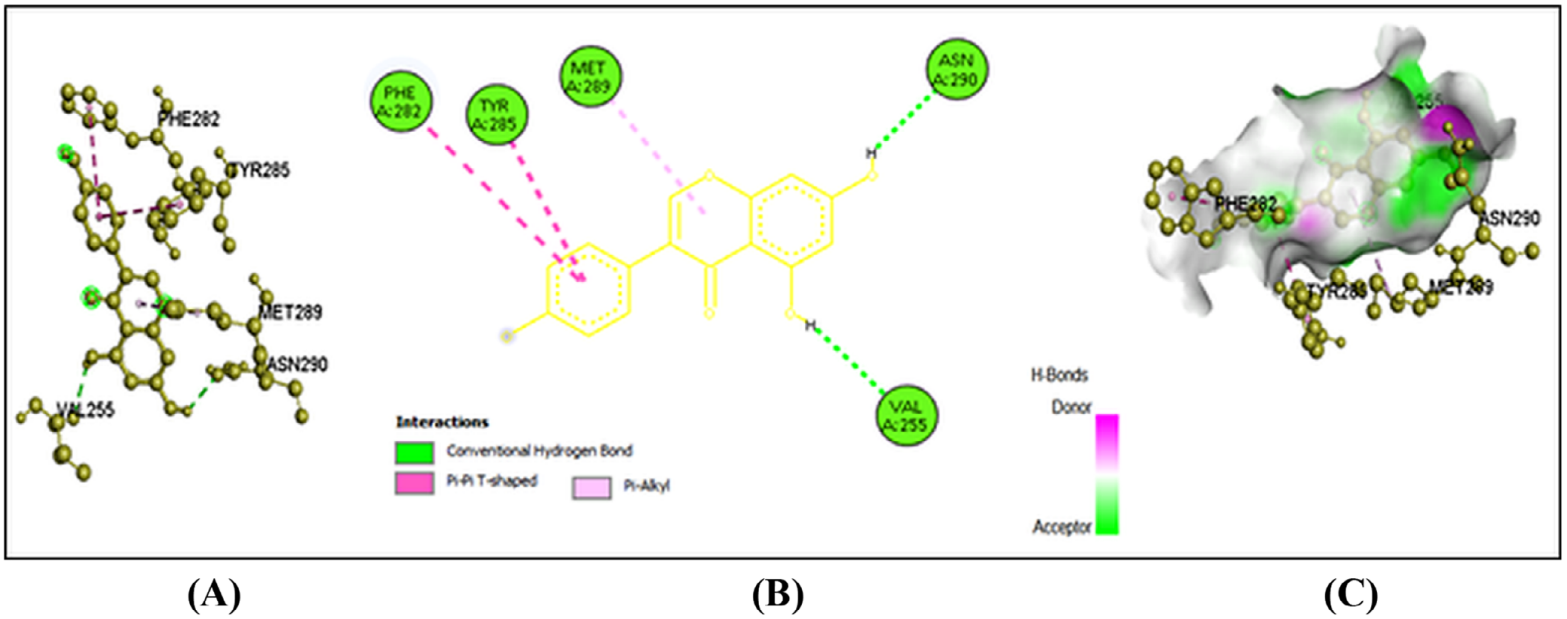
Molecular docking of genistein with gamma‐carboxylase: (A) 3D interaction, (B) 2D structural interactions, and (C) hydrogen‐bonding surface.

**FIGURE 12:**
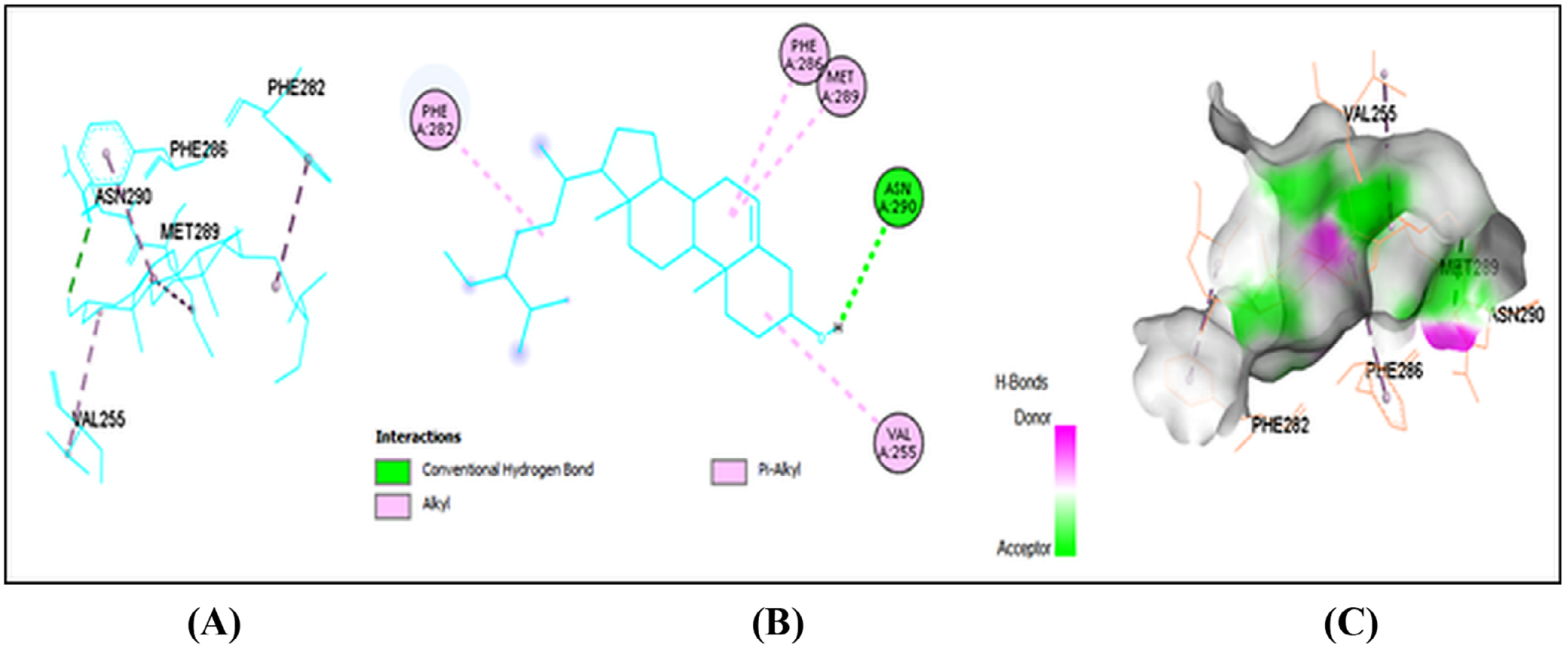
Molecular docking of β‐sitosterol with gamma‐carboxylase: (A) 3D interaction, (B) 2D structural interactions, and (C) hydrogen‐bonding surface.

**FIGURE 13:**
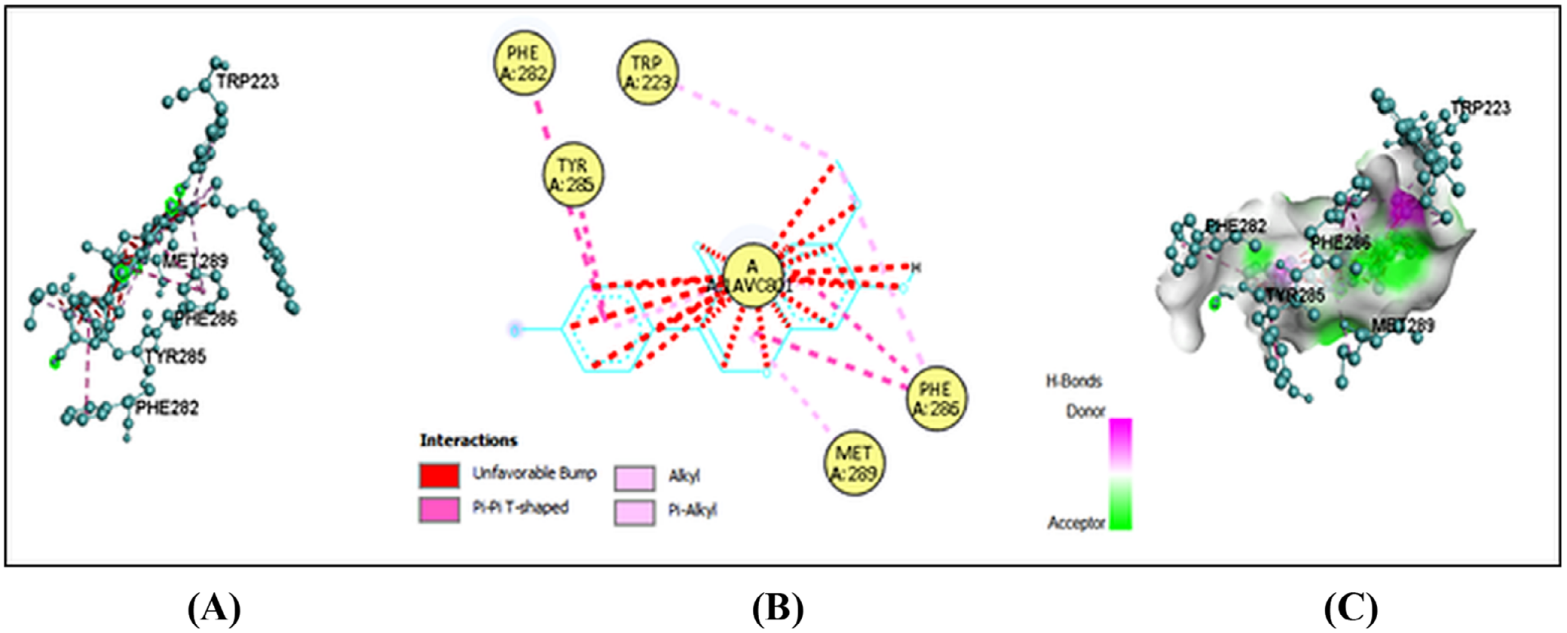
Molecular docking of Glycitein with gamma‐carboxylase (A) 3D interaction, (B) 2D structural interactions, and (C) hydrogen‐bonding surface.

**FIGURE 14:**
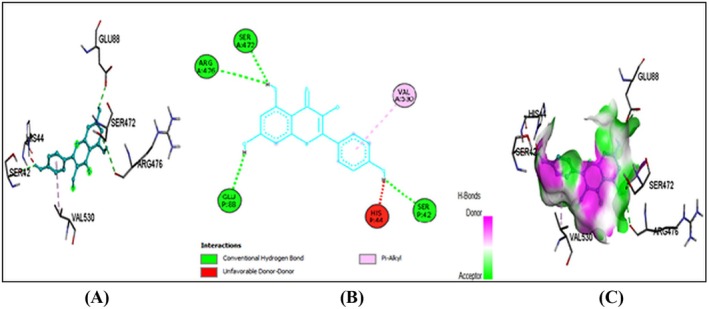
Molecular docking of kaempferol with gamma‐carboxylase: (A) 3D interaction, (B) 2D structural interactions, and (C) hydrogen‐bonding surface.

**FIGURE 15:**
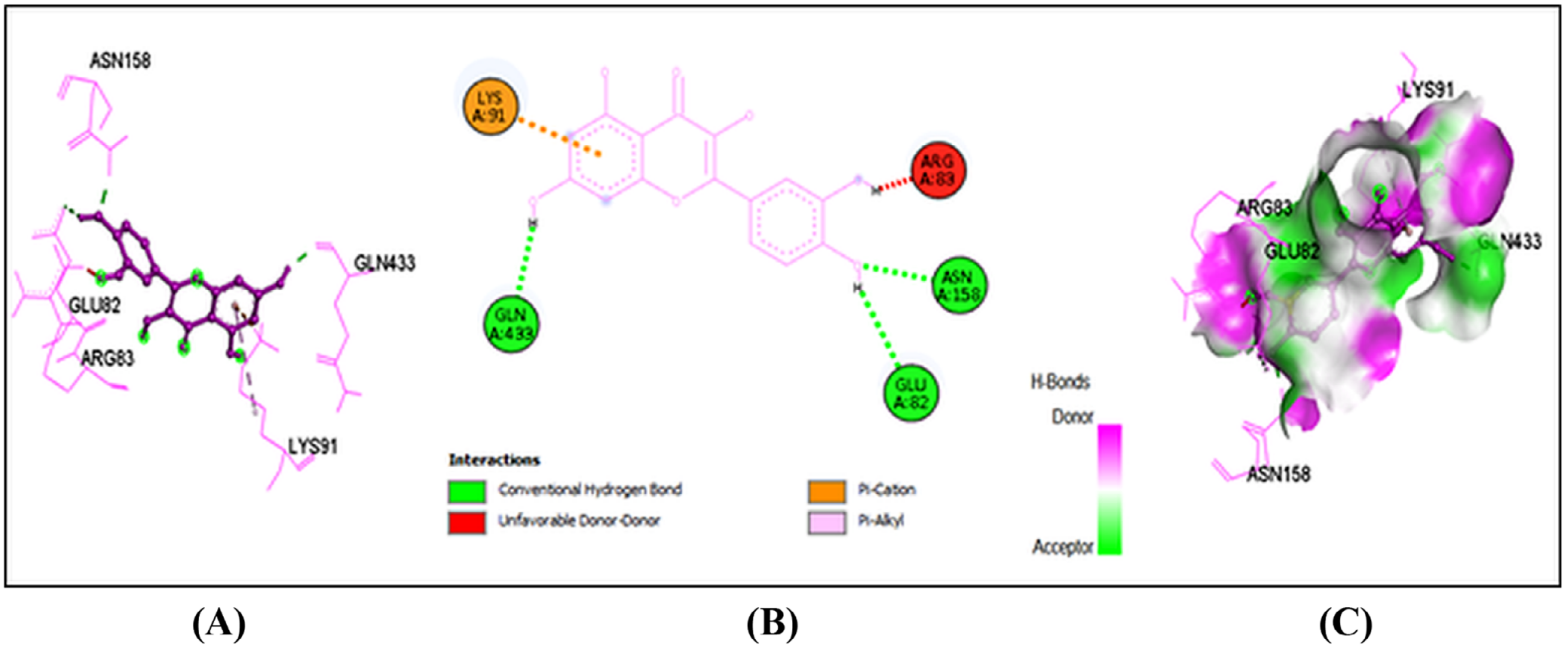
Molecular docking of quercetin with gamma‐carboxylase: (A) 3D interaction, (B) 2D structural interactions, and (C) hydrogen‐bonding surface.

Molecular docking analysis was performed to assess the anti‐osteoporosis potential of bioactive compounds from soybean and pea, including β‐sitosterol, genistein, quercetin, glycitein, and kaempferol. The 2D and 3D binding interactions of the bioactive compounds with the target protein, including bond distances, interaction categories, specific types of interactions, and binding energies, are presented in Figures 11, 12, 13, 14, 15 and Table 4. The results demonstrated that β‐sitosterol showed the strongest binding affinity (−9.0 kcal/mol) as compared with genistein (−8.3 kcal/mol), quercetin (−8.1 kcal/mol), glycitein, and kaempferol (−7.6 kcal/mol), respectively. Moreover, interactions such as hydrogen bonding, hydrophobic, conventional hydrogen bonding, Pi‐Pi T‐shaped, Pi‐Alkyl, Alkyl, Pi‐Cation, and Pi‐Donor Hydrogen Bond were identified between bioactive components (genistein, β‐sitosterol, glycitein, kaempferol, and quercetin) and gamma‐carboxylase (Figures 12, 13, 14).

**TABLE 4:** Present interactions of bioactive compounds with protein residues, showing distances, types, and binding energies.

Compound	Name	Distance (Å)	Category	Types	Energy
Genistein	VAL 255	2.54343	Hydrogen bond	Conventional Hydrogen bond	−8.3
	PHE 282	5.5572	Hydrophobic	Pi‐Pi T‐shaped	
	ASN 290	2.46195	Hydrophobic	Conventional Hydrogen bond	
	MET 289	4.78267	Hydrophobic	Pi‐Alkyl	
	TRY 285	4.99813	Hydrophobic	Pi‐Pi T‐shaped	
β‐sitosterol	PHE 282	4.43201	Hydrophobic	Pi‐Alkyl	−9
	PHE 286	4.93706	Hydrophobic	Pi‐Alkyl	
	MET 289	4.73746	Hydrophobic	Alkyl	
	VAL 255	5.51703	Hydrophobic	Alkyl	
	ASN 290	2.97082	Hydrogen bond	Conventional Hydrogen bond	
Glycitein	PHE 282	5.50776	Hydrophobic	Pi‐Pi T‐shaped	−7.6
	PHE 286	5.04688	Hydrophobic	Pi‐Pi T‐shaped	
	TYR 285	5.15312	Hydrophobic	Pi‐Pi T‐shaped	
	TRP 223	5.00411	Hydrophobic	Pi‐Alkyl	
Kaempferol	SER 42	2.32159	Hydrogen bond	Conventional Hydrogen bond	−7.6
	SER 472	2.40602	Hydrogen bond	Conventional Hydrogen bond	
	ARG 476	2.8409	Hydrogen bond	Conventional Hydrogen bond	
	GLU 88	2.49882	Hydrogen bond	Conventional Hydrogen bond	
	VAL 530	5.14006	Hydrophobic	Pi‐Alkyl	
Quercetin	ASN 158	1.75826	Hydrogen bond	Conventional Hydrogen bond	−8.1
	GLU 82	2.37249	Hydrogen bond	Conventional Hydrogen bond	
	GLN 433	1.93693	Hydrogen bond	Conventional Hydrogen bond	
	LYS 91	2.80705	Hydrogen bond; Electrostatic	Pi‐Cation; Pi‐Donor hydrogen bond	

## 4 Discussion

Osteoporosis is a crucial bone disorder characterized by bone loss and structural decline. In this research, soybean and pea protein isolates were prepared by alkaline extraction and isoelectric precipitation, which is a widely used method for protein isolation. This process will remove most of the carbohydrates and increase the protein content of the isolates. Thus, the soybean and pea protein isolates obtained should have fewer residual carbohydrates (polysaccharides) content has been described by prior studies on the preparation of protein isolates (Rout et al. 2024). Moreover, qualitative chemical tests (Biuret and Ninhydrin reactions) were conducted to identify the occurrence of the proteins and amino acids in the prepared isolates, in line with standard biochemical procedures (Godlewska et al. 2022; Morano et al. 2025). Our findings align with those of Gorissen et al. (2018), who reported that both isolates are at the highest level of glutamic and aspartic acids, and the lowest level of sulfur‐containing amino acids. The amino acid content of PPI was also greater than that of SPI, as it was rich in arginine and lysine. Glutamic acids and aspartic acids have a role in bone metabolism and osteoclast activity (Hu et al. 2024). Furthermore, lysine is involved in calcium absorption and collagen synthesis, and arginine is involved in nitric oxide synthesis, which aids in bone repair. Therefore, these amino acids may help support bone health (Liang et al. 2024).

These protein isolates have potential benefits in preventing osteoporosis and supporting bone health. In the current study, the bone restoring potential of soybean and pea protein isolates was evaluated. The in vivo findings indicated that T_3_ improves and normalizes feeding and drinking behavior in rats. An efficacy study showed that a significant reduction was observed in the feed and water intake of osteoporotic rats, but feed and water intake improved in the treatment groups. Our findings showed similar trends to those observed by Malkawi et al. (2018), who stated that regular administration of dexamethasone decreases appetite and energy intake. The present results are in line with those of Ali et al. (2015), who found that the efficiency of feed in Sprague Dawley rats was considerably increased by pressurized soy protein isolates. However, a mixture of SPI and PPI improved feed and water intake because protein molecules are more accessible for digestion, which enhances nutrient absorption and supports overall growth and development, especially observed in the high dose of the SPI and PPI mixture. These findings indicate that T_3_ improved body weight in the experimental conditions. Our findings showed similar trends to those observed by Malkawi et al. (2018), who determined that regular administration of dexamethasone led to weight loss that included decreased muscle mass, along with growth suppression and reductions in bone and organ mass. Furthermore, just one dose of dexamethasone caused a significant decrease in body weight, primarily from the loss of sodium and water (Jahng et al. 2008). However, a mixture of SPI and PPI improved body weight due to regulating appetite and improving metabolism, especially observed in the high dose of the SPI and PPI mixture. The present outcomes are in line with those of Ali et al. (2015), who found that pressurized soy protein isolates significantly increased body length, weight, and feed efficiency in Sprague Dawley rats. Our findings showed a similar trend to that observed by Wei et al. (2021), who reported that malnourished rats' body weight and physiological state were considerably improved by consuming soy protein. However, another study showed that intake of 40 g of soy protein isolates daily for 12 weeks resulted in a significant increase in muscle strength among postmenopausal women (Shenoy et al. 2013), consistent with their anabolic role. The anabolic activity of pea protien may have been similar to that of casein, partly because of its high digestibility and amino acid pattern, in particular because of its essential leucine content, which is considered a key amino acid for the synthesis of muscle protein (Salles et al. 2021). Our results are consistent with those of Urbano et al. (2003), who showed that by increasing digestibility and decreasing inhibitory factors, hydrothermal treatment of pea flour greatly increased the protein capacity to support growth.

The improvement in the hematological profile in the present study may be attributed to soy's rich amino acid profile, which supports hemoglobin synthesis. Soy isoflavones can also help reduce oxidative stress in bone marrow, and leucine‐rich pea protein could facilitate protein synthesis and nutrient utilization. Similar alterations were observed as reported by Bosland et al. (2021), showing that taking dietary supplements of 20 g/day of SPI for two years caused the serum ferritin level to double and hemoglobin and hematocrit levels to slightly increase when compared to casein and soy supplementation, reflecting enhanced erythropoiesis.

On the other hand, liver function enzymes, including ALT, AST, ALP, and bilirubin, were decreased in the treatment groups, likely due to the beneficial effects of soy and pea proteins in improving liver function by supporting enzyme regulation, metabolic activity, and detoxification processes. Similar to our results, according to research by Nabih et al. (2023), eating soy isoflavones and genistein significantly decreased the level of ALT enzymes. Hakkak et al. (2018) revealed that obese rats fed soy had decreased serum AST and ALT levels compared with controls fed casein. In another study, rats given a diet consisting of a blend of salmon and soybean protein isolates for 12 weeks showed increased levels of bilirubin and serum alkaline phosphatase (Maditz et al. 2015). Additional evidence supports SPI's hepatoprotective effects in both human and animal studies (Liu et al. 2023; Eftekhari et al. 2013), which align with our results. Within this study, normalization of renal biomarkers may result from soy‐mediated anti‐inflammatory signaling, while pea protein's arginine content may enhance renal perfusion and nitric oxide bioavailability, supporting nephroprotection. These effects collectively suggest that soy and pea proteins positively influence kidney function by reducing renal workload and improving overall metabolic health. These results align with those of Ferraz Carbonel et al. (2022), who indicated that soy isoflavones reversed renal injury by decreasing KIM‐1 expression and collagen deposition, associated with PPAR‐γ activation. Furthermore, Sankar et al. (2019) demonstrated that ovariectomy markedly raised serum urea, creatinine, and uric acid, indicating impaired kidney function. Therefore, consuming soy isoflavones in the diet considerably reduced these parameters, restoring kidney function. Overall, administration of soy isoflavones alleviated metabolic alterations caused by ovariectomy.

The reduction in serum osteocalcin, calcium and phosphorus levels, along with elevated inflammatory markers in the dexamethasone‐treated group, supports successful induction of osteoporosis related disturbances. The levels of serum osteocalcin, Ca, and P increased in the treatment groups and supported bone health. This may be because dexamethasone suppressed osteocalcin and disrupted the level of Ca and P homeostasis. However, SPI and PPI enhanced the absorption of minerals, leading to a more positive balance for bone health. SPI and PPI significantly increased osteocalcin and ALP, consistent with their osteogenic activity. Soy isoflavones act as phytoestrogens, enhancing Runx2 expression and stimulating osteoblast differentiation (Wang et al. 2022), while pea‐derived peptides such as LRW activate Akt signaling and promote mineralization (Arora et al. 2020). Additionally, soy isoflavones reduced urinary Ca levels, with a more notable effect noted in soy protein isolates compared with soy milk (Spence et al. 2005). Comparable to Gallagher et al. (2004), where SPI increased osteocalcin, our findings suggest improved bone formation. In another study, pea protein demonstrated a moderate and statistically nonsignificant effect relative to water, resembling those of whey proteins (Nieman et al. 2020). Earlier studies demonstrated that soybean and fermented soy flour 10% and 20% decreased osteoclast count in rat tibia bones, implying a protective role against osteoporosis (Wresdiyati et al. 2021). In their study, Almajwal et al. (2019) evaluated bio‐nano‐encapsulated vitamin D delivered via sonication and pH‐shifted pea protein isolate, and observed that vitamin D‐deficient rats showed improvements associated with modification in serum Ca, P, PTH, and ALP levels. Our findings demonstrated that both SPI and PPI mixtures reduce CRP and ESR levels. Dexamethasone exposure is known to elevate systemic inflammation, as reflected by increased ESR and CRP. Moreover, both SPI and PPI exhibit notable antioxidative and inflammation‐modulating properties. Soy isoflavones, in particular, inhibit NF‐kB signaling and reduce IL‐6 and TNF‐alpha production (Asbaghi et al. 2020), while PPI hydrolysates strongly suppress LPS‐stimulated TNF‐alpha and IL‐6 secretion in macrophages and reduce inflammatory responses in mice, supporting a peptide‐mediated anti‐inflammatory mechanism for PPI (Ndiaye et al. 2012).

A previous study has demonstrated that dexamethasone administration induces bone loss and structural deterioration in skeletal tissue. Similar histopathological alterations were observed in the present study, confirming the successful induction of osteoporosis in the experimental rats (Shomali et al. 2016). The osteoporotic group exhibited marked histological changes, including trabecular thinning, enlarged marrow cavities, and disruption of normal bone architecture. These findings are characteristic of glucocorticoid‐induced osteoporosis and reflect an imbalance between bone formation and bone resorption (Briot and Roux 2015). The benefits of the mixture of SPI and PPI on bone histopathology align with earlier findings regarding the protective effects of soybean protein isolates on normal architecture and reducing glucocorticoid‐induced bone damage in osteoporotic rats. The beneficial effects of SPI on bone health might be related to its bioactive compounds, such as isoflavones that have been demonstrated to inhibit bone loss and promote bone maintenance. Messina (2014) also noted similar observations regarding the positive effect of soy foods on bone health. In addition to their beneficial effects on bone tissue, SPI and PPI preserved normal renal morphology. The glomerular architecture was maintained and tubular degeneration was decreased in the treated groups compared with the osteoporotic group. These results are consistent with previous studies that showed soy isoflavones can help maintain healthy kidney tissue structure (Misiakiewicz‐Has et al. 2024).

Notably, the greater changes that have been seen in T_3_ than in T_2_ would imply that there is a dose‐related response biologically. Past studies have indicated that soybean and pea protein isolates are abundant in essential amino acids and can be used to produce bioactive peptides that have anabolic, osteogenic, and anti‐inflammatory effects (Alleza et al. 2025; Gan et al. 2025). Increase intake of these protein isolates could improve the physiological and biochemical parameters by stimulating signaling pathways related to bone metabolism, antioxidant activity and immune modulation. These effects can promote bone development, enhance antioxidant enzyme activity, and regulate inflammatory mediators, which can help to reduce inflammation (Kedzia et al. 2023; Franca‐Oliveira et al. 2023).

The molecular docking findings align with those of Zaklos‐Szyda et al. (2020), who found that soy isoflavones such as genistein, daidzein, and glycitein have a high binding affinity for RANKL, implying interference with osteoclast‐related signaling. Furthermore, Hu et al. (2022) demonstrated that quercetin could regulate inflammatory cytokines involved in osteoporosis, related to estrogen signaling and oxidative stress. Ruangsuriya et al. (2020) reported increased osteoblast differentiation markers such as ALP and OPG and decreased osteoclastogenic signaling (RANKL) after treatment with the flavonoid‐rich fraction. Moreover, Tang et al. (2022) reported that kaempferol regulates numerous markers related to bone metabolism, including the PI3K‐Akt, MAPK, and estrogen pathways. This compound demonstrated high binding activity to core proteins that regulate bone remodeling, supporting its capability to act as a multi‐target natural intervention for age‐related osteoporosis. The molecular docking results in the present study indicated that bioactive compounds have a good binding affinity for gamma‐carboxylase, which indicates a possible interaction with bone‐related regulatory mechanisms. However, the findings are preliminary and need to be explored further for validation.

## 5 Limitations

This study has some limitations, including the absence of a positive control group receiving standard anti‐osteoporotic treatments, which limited direct comparison of the efficacy of the mixture of SPI and PPI. Furthermore, although molecular docking indicates possible mechanisms of action, these proposed mechanisms were not experimentally validated through pathway analysis, inhibition assays, gene expression analysis, and knockout experiments. Moreover, another limitation is the determination of IGF‐1, a key factor involved in bone formation. Therefore, future studies should incorporate an appropriate positive control group and mechanistic investigations, along with assessment of IGF‐1 to confirm the underlying biological pathways and strengthen the findings.

## 6 Conclusion

The present study demonstrates that both SPI and PPI possess high amino acid contents. PPI has a more content of amino acids such as arginine, lysine, and leucine as compared with SPI. Both isolates have lower levels of sulfur‐containing amino acids, and the tryptophan values are similar in both isolates. SPI and PPI showed significant improvements observed in the hematological profile, and protective effects on liver enzymes and renal biomarkers, and also maintain serum osteocalcin, Ca, and P, as well as decreased inflammatory markers CRP and ESR, respectively. Furthermore, histopathological examination showed that the mixture of SPI and PPI improved bone structure and reduced osteoporosis‐associated tissue alterations in rats. Additionally, normal liver and kidney morphology was preserved, suggesting potential effects. Notably, the T_3_ treatment dose showed the greatest improvement in osteoporosis‐associated biochemical, inflammatory biomarkers, and histopathologically. Additionally, molecular docking provided preliminary computational insight into the possible interactions of bioactive compounds with bone‐related targets. From a future perspective, human trials are needed to establish the efficacy and safety of SPI and PPI for osteoporosis management.

## Author Contributions

**Muhammad Tauseef Sultan:** project administration. **Shehnshah Zafar:** writing – review and editing. **Adnan Amjad:** conceptualization, supervision. **Rabia Mehboob:** writing – review and editing. **Hassan Raza:** writing – review and editing, visualization. **Hafiza Humaira Yasmeen:** formal analysis, writing – original draft. **Zafarullah Muhammad:** formal analysis. **Khurram Afzal:** resources, project administration. **Muhammad Israr:** writing – review and editing, software. **Ahmad Mujtaba Noman:** writing – review and editing. **Entessar Mohammad Al Jbawi:** writing – review and editing, validation.

## Funding

The authors have nothing to report.

## Conflicts of Interest

The authors declare no conflicts of interest.

## Data Availability

The data that support the findings of this study are available from the corresponding author upon reasonable request.
